# The effect of varying irrigation flow rate during irrigated radiofrequency ablation on optimising lesion shape

**DOI:** 10.1093/europace/euad321

**Published:** 2023-10-27

**Authors:** Rachael E Redgrave, Anna Walaszczyk, Micheylla Kusumaning Dewi, Maria Camacho Encina, Jilek Clemens, Ruairidh Matrin, Gavin D Richardson, Moloy Das

**Affiliations:** Vascular Medicine and Biology Theme, Biosciences Institute, Newcastle University, International Centre for Life, Times Square, Newcastle upon Tyne, NE1 4EP, UK; Vascular Medicine and Biology Theme, Biosciences Institute, Newcastle University, International Centre for Life, Times Square, Newcastle upon Tyne, NE1 4EP, UK; Vascular Medicine and Biology Theme, Biosciences Institute, Newcastle University, International Centre for Life, Times Square, Newcastle upon Tyne, NE1 4EP, UK; Vascular Medicine and Biology Theme, Biosciences Institute, Newcastle University, International Centre for Life, Times Square, Newcastle upon Tyne, NE1 4EP, UK; Peter-Osypka Heart Centre Munich, Internistisches Klinikum München Süd, Am Isarkanal 36, 81379 München, Munich, Germany; Technical University of Munich, Arcisstraße 21, 80333 München, Munich, Germany; Department of Cardiology, The Newcastle Upon Tyne Hospitals NHS Foundation Trust, Freeman Hospital, Freeman Rd, High Heaton, Newcastle upon Tyne, NE7 7DN, UK; Vascular Medicine and Biology Theme, Biosciences Institute, Newcastle University, International Centre for Life, Times Square, Newcastle upon Tyne, NE1 4EP, UK; Vascular Medicine and Biology Theme, Biosciences Institute, Newcastle University, International Centre for Life, Times Square, Newcastle upon Tyne, NE1 4EP, UK; Department of Cardiology, The Newcastle Upon Tyne Hospitals NHS Foundation Trust, Freeman Hospital, Freeman Rd, High Heaton, Newcastle upon Tyne, NE7 7DN, UK

## Abstract

**Graphical Abstract:**
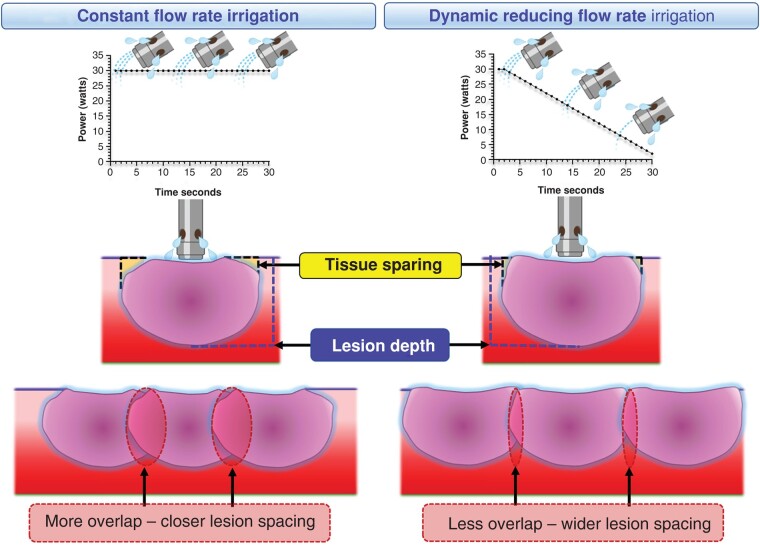

Radiofrequency ablation commonly requires the creation of a contiguous line of ablation lesions, with even small gaps potentially leading to breakthrough conduction.^1^ Lesion spacing is dependent on lesion size, which is influenced by factors including power delivery, tissue contact and application duration, but also lesion shape as this determines the degree of lesion overlap required to achieve contiguity. Irrigated catheters are the standard tool for radiofrequency ablation in the left heart. However, lesions created by irrigated catheters demonstrate a ‘tear-drop’ shape due to endocardial sparing.^2^ Manufacturer-recommended flow rates of 17 mL/min for ≤30 W and 30 mL/min for >30 W appear to be relatively arbitrary and there are scant data to support these figures or this dichotomy. Equally, a low flow rate of 2 mL/min may be insufficient to provide adequate lesion depth in thicker tissue.^3^ We, therefore, assessed if varying the irrigation flow rate during ablation could optimise lesion shape by minimising endocardial sparing while maintaining lesion depth, in turn improving the efficiency of ablation line delivery (see Supplementary material online, *Figure S1*).

As detailed in the Supplementary Methods, ablations were performed at 30 W for 30 s using an *ex vivo* model and six different irrigation flow-rate protocols (*Table 1* and Supplementary material online, *Figure S2*). Perpendicular catheter orientation was used throughout for consistency as catheter angulation can influence lesion shape.

**Table 1:** Comparison of ablation protocols. *Protocol key*: Protocol A17 – Fixed irrigation rate: 17 mL/min. Protocol A30 – Fixed irrigation rate: 30 mL/min. Protocol B – Continuous reduction in the irrigation rate: 30 mL/min (first 2 s), reducing by 1 mL/min per second to 2 mL/min. Protocol C – Continuous increase in the irrigation rate: 2 mL/min (first 2 s), increasing by 1 mL/min per second to 30 mL/min. Protocol D – Stepwise reduction in the irrigation rate: 30 mL/min (first 10 s), 16 mL/min (next 10 s), 2 mL/min (last 10 s). Protocol E – Stepwise increase in the irrigation rate: 2 mL/min (first 10 s), 16 mL/min (next 10 s), 30 mL/min (last 10 s)

Power (W)	Protocol	Total no. lesions	Max tip temp (⁰C)	Terminated (ET)	Steam pop	Other reasons	Measurable lesions
**30**	A17	12	30.00 ± 1.13	0	0	0	12
	A30	20	26.75 ± 0.91	0	1 (5%)	3	16
	B	20	45.35 ± 4.97	0	1 (5%)	4	15
	C	20	41.00 ± 3.13	0	1 (5%)	2	17
	D	10	60.30 ± 8.23	8 (80%)	0	0	2
	E	10	51.70 ± 4.9	2 (20%)	0	1	7
**20**	A17	14	27.93 ± 1.07	0	0	0	14
	B	14	44.07 ± 6.28	0	0	0	14
**40**	A30	13	27.15 ± 0.99	0	1 (7.7%)	0	12
	B	13	49.64 ± 7.18	1 (7.7%)	2 (15.4%)	0	10

ET, excessive temperature.

All dynamic flow-rate protocols (B–E) produced significantly higher mean tip temperatures than fixed flow-rate protocols (*Table 1*). Recorded numbers of steam pops were comparable between all protocols. Protocols D and E resulted in premature cut-offs of radiofrequency delivery due to excessive tip temperatures (>50°C) and were, therefore, excluded from subsequent analysis.

Morphometric assessment demonstrated that continuous reduction in flow-rate (Protocol B) significantly decreased endocardial sparing compared to fixed flow-rate protocols (Protocols A17 and A30) and trended towards a reduction compared to a continuous increase in flow rate (Protocol C) (*Figure 1A* and *B*). Surface diameter was also significantly larger for Protocol B compared to Protocol A17 and importantly, lesions produced with Protocol B demonstrated no decrease in depth or volume (*Figure 1B*).

**Figure 1:**
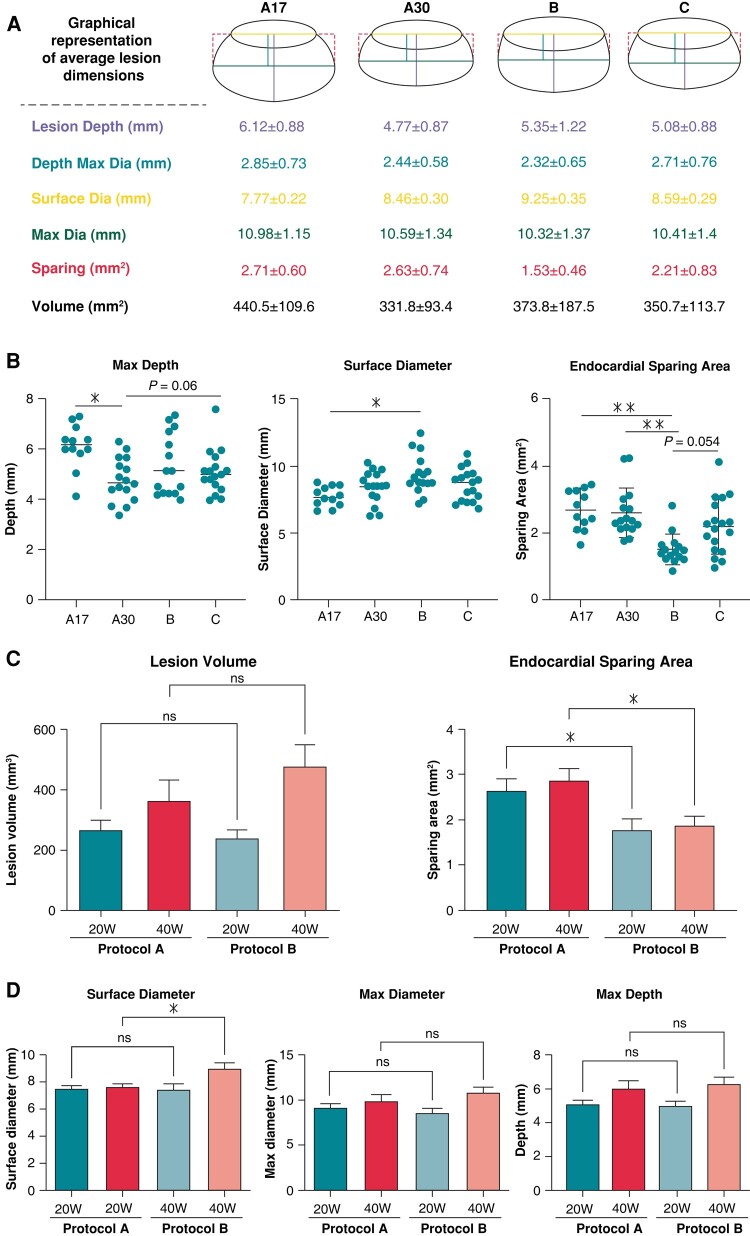
*A* **)** Graphical representation to scale of lesions created with protocols A17, A30, B and C. All ablations were performed at 30 W. Values represent mean ± SD. *B***)** Graphs of calculated mean measurements for Protocols A17, A30, B and C. *C***)** Lesion volume and endocardial sparing area from fixed flow (Protocol A) or continuous reduction in flow rate (Protocol B) at 20 W and 40 W. *D***)** Surface diameter, max diameter, and max depth of lesions using Protocol A or B at a power of 20 or 40 W. For all data *P < 0.05, **P < 0.01.

Given the possible utility of Protocol B to improve ablation efficiency, we assessed the influence of power (20 or 40 W) on this protocol. Maximum tip temperature, ablations terminated due to excessive temperature and the number of steam pops are shown in *Table 1*. At all powers, maximum tip temperature was significantly higher for Protocol B compared to continuous flow (Protocol A) (*Table 1*). Endocardial sparing area was significantly reduced with for Protocol B at both powers while lesion volume was maintained (*Figure 1C* and *D*).

We then compared dynamic reduction in flow-rate ablation to continuous flow in an *in vivo* porcine model. A total of 78 lesions (41 atrial; 37 ventricular) were performed in four animals (see Supplementary material online, *Table S1*). Despite treatment with IV Amiodarone and, in 1 case, IV Lignocaine also, 13 of 37 (35%) of ventricular lesions resulted in VT or VF, requiring premature termination of energy delivery and in most instances DC cardioversion, resulting in potential map shift. Maximum recorded tip temperature was significantly higher for Protocol B than for fixed flow rate in both the atrium and the ventricle. Only one ventricular lesion using Protocol B was prematurely terminated at 29 s due to excessive temperature (55°C). No steam pops occurred and there was no evidence of char on the catheter tip on visual inspection for either protocol.

Ventricular lesions displayed no significant difference in electrogram amplitude reduction or local impedance (LI) drop with ablation between the two protocols. Atrial fixed flow-rate lesions had a significantly higher mean starting LI, indicating greater catheter–tissue coupling, and, as would be expected as starting LI is predictive of LI drop, mean and % LI drop were also significantly greater (see Supplementary material online, *Table S1*). All four animals recovered from anaesthesia and survived until sacrifice after 11–12 days. Due to curtailed lesions and map shifts following DC cardioversion affecting sequential lesion placement, it was not possible to perform morphometric analysis on the *in vivo* lesions.

To create contiguity, current clinical data suggest that lesions should be placed <5–6 mm apart,^4,5^ recommendations which are supported by our data, and previous findings.^2^ However, if the maximal diameter could be extended to the endocardial surface with no loss of lesion depth or volume, lesions could potentially be placed approximately 8–10 mm apart, thereby markedly reducing the number of lesions required to complete an anatomical line (see Supplementary material online, *Figure S1*). While studies have suggested adopting a low irrigation flow rate such as 2–5 mL/min to reduce endocardial sparing and increase surface diameter, low irrigation flow rates were also associated with reduced lesion depth at lower power settings,^3^ and excessively high tissue temperatures and the occurrence of steam pops at higher powers.^6,7^

These factors limit the ability to utilise fixed low flow rate in clinical practice. Here, we show that continuous reduction in the irrigation flow rate from 30 to 2 mL/min over 30 s resulted in a more optimal lesion shape with significantly reduced endocardial sparing compared to a fixed irrigation flow rate. Notably, this was not at the expense of lesion depth or volume and no significant increase in steam pops was observed. This, therefore, raises the potential for wider lesion spacing by using continuous reduction in irrigation flow rate during point-by-point radiofrequency ablation.

In a large animal model, mean tip temperature recorded for lesions created with continuous reduction in flow rate was significantly higher than for fixed-rate irrigation, but importantly, was not excessive at approximately 43°C. With the flow rate falling progressively to 2  mL/min by the end of the 30-s application, the temperature at the tip–tissue interface would be expected to rise towards temperatures typically seen with solid-tip irrigation, which explains the reduction in endocardial sparing. However, because the period of low flow rate is not prolonged, the excessive temperatures seen in the previous studies referenced above were not reached. While additional *in vivo* and clinical studies are required to demonstrate the clinical utility of these data, our study suggests that dynamic flow-rate protocols are safe and may improve irrigated radiofrequency ablation efficiency.

## Supplementary Material

euad321_Supplementary_Data

## Data Availability

The data underlying this article will be shared on reasonable request to the corresponding author.
