# Supplementary material for: The effect of varying irrigation flow rate during irrigated radiofrequency ablation on optimising lesion shape
Source: Europace. 2023 Oct 27;26(1):euad321. doi: 10.1093/europace/euad321 (PMC10754152; doi:10.1093/europace/euad321)
Supplement: euad321_Supplementary_Data [file euad321_supplementary_data.zip › Supplementary Data.docx]

**Supplementary Data**


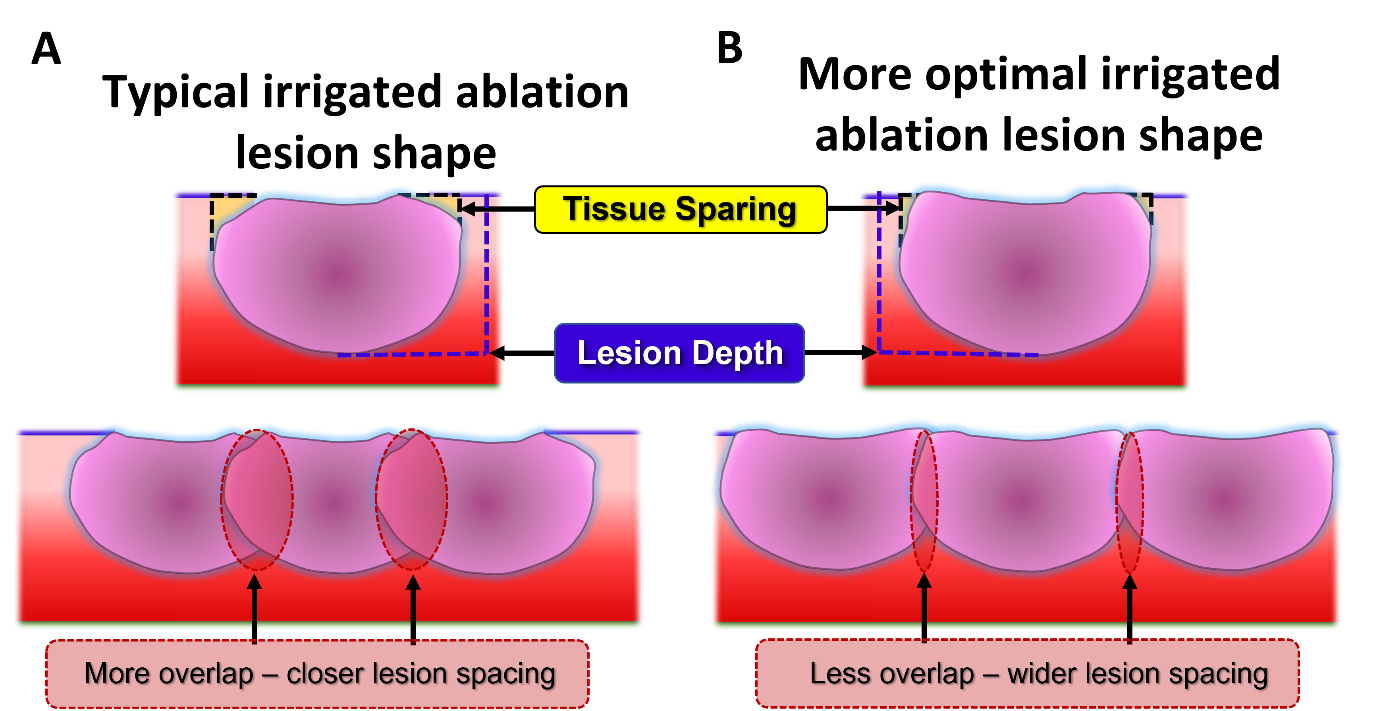


**Supplementary Figure 1: A)** Typical irrigated ablation lesion shape with illustration of the degree of overlap required to create an ablation line that is contiguous at the endocardial surface – wider lesion spacing would result in endocardial gaps. **B)** More optimal irrigated ablation lesion shape, with reduced endocardial sparing, allowing for wider lesion spacing while maintaining endocardial contiguity.


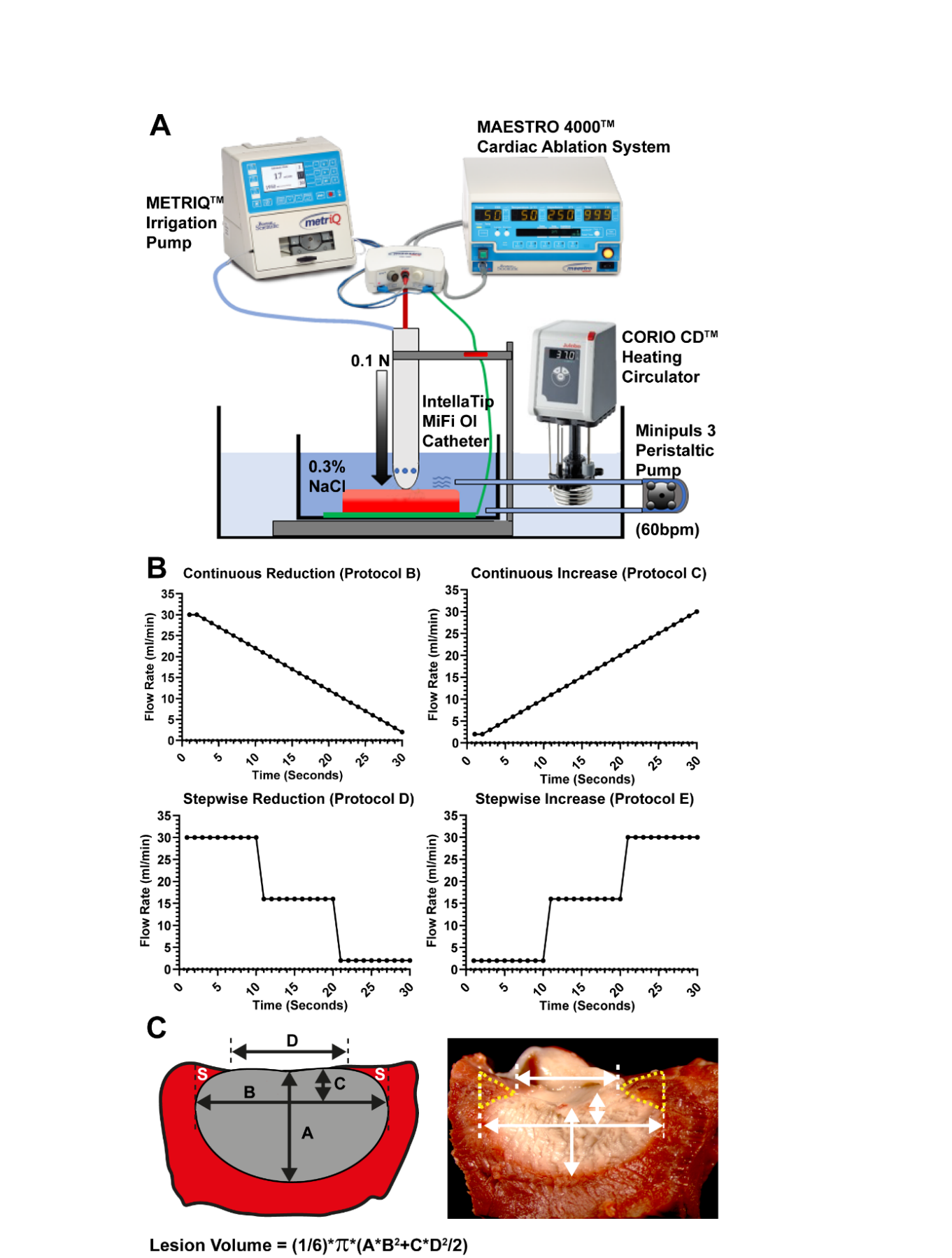


**Supplementary Figure 2. *Ex vivo* set up and geometry assessment**. ***A*:** *Ex vivo* setup. ***B*:** Graphs showing flow-rate against ablation time for each dynamic flow-rate protocol (Protocols B-E). ***C*:** Diagram and example of lesion measurement protocol: maximum depth (A), maximum diameter (B), depth at maximum diameter (C), and lesion surface diameter (D). Volume calculated as lesion volume = (1/16*π*(A*B^2^)A*B^2+^D^2^/2). S = endocardial sparing area (yellow-dotted area in example image).

|  |  | Fixed irrigation rate | Reducing irrigation rate | *P* value |
| --- | --- | --- | --- | --- |
| Atrial lesions (n=41) | Max tip temperature (°C) | 33.96 ± 0.84 | 42.50 ± 2.51 | <0.0001 |
|  | Starting amplitude (mV) | 2.08 ± 1.29 | 1.34 ± 0.41 | 0.052 |
|  | Amplitude decrease (mV) | 0.71 ± 0.93 | 0.58 ± 0.33 | 0.613 |
|  | Starting LI (Ω) | 171.30 ± 18.59 | 152.90 ± 10.01 | 0.002 |
|  | LI drop (Ω) | 30.07 ± 13.98 | 18.00 ± 7.67 | 0.006 |
|  | %LI drop (%) | 17.17 ± 6.02 | 11.80 ± 4.10 | 0.008 |
| Ventricular lesions (n=24) | Max tip temperature (°C) | 34.42 ± 0.515 | 43.83 ± 4.53 | <0.0001 |
|  | Starting amplitude (mV) | 2.98 ± 2.05 | 2.67 ± 1.14 | 0.647 |
|  | Amplitude decrease (mV) | 1.10 ± 1.31 | 1.06 ± 0.64 | 0.924 |
|  | Starting LI (Ω) | 156.10 ± 55.83 | 138.00 ± 28.06 | 0.327 |
|  | LI drop (Ω) | 25.75 ± 30.87 | 18.000 ± 13.48 | 0.434 |
|  | %LI drop (%) | 13.40 ± 10.28 | 11.86 ± 7.62 | 0.680 |

**Supplementary Table 1:**
