# Supplementary material for: The effect of varying irrigation flow rate during irrigated radiofrequency ablation on optimising lesion shape
Source: Europace. 2023 Oct 27;26(1):euad321. doi: 10.1093/europace/euad321 (PMC10754152; doi:10.1093/europace/euad321)
Supplement: euad321_Supplementary_Data [file euad321_supplementary_data.zip › Supplementary Methods.pdf]

Supplementary Methods

Ex vivo tissue preparation

Ablations were performed in porcine hearts as previously described by Guerra *et al* (**Supplementary Figure 2A**).[2] Sections of left ventricular tissue at a size of 6x3cm were placed on an adhesive grounding pad attached to an indifferent lead (Valleylab, Covidien, Mansfield, MA) in 0.3% NaCl (to mimic the resistivity of human blood) at 37°C. All ablations were delivered with a constant force of 0.1N (10g). A pulsatile flow of 60bpm was directed at the ablation electrode-tissue interface using a Minipuls 3 peristaltic pump (Gilson, Middleton, USA).

Ex vivo ablation

All ablations were performed with an IntellaTip MiFi open-irrigated catheter (Boston Scientific, Marlborough, MA), which incorporates a 4.5mm open-irrigated tip. A MAESTRO 4000™ Cardiac Ablation System (Boston Scientific, Marlborough, MA) was used to control power, maximum tip temperature, impedance, and duration. As all of the varying flow rate protocols involved a period of irrigation at 2ml/min, which is essentially equivalent to solid-tip radiofrequency ablation, the maximum temperature was set at 50°C. A METRIQ™ Irrigation Pump (Boston Scientific, Marlborough, MA) was used to adjust catheter flow rates. Maximum electrode tip temperature, occurrence of steam pops and terminated ablations due to excessive temperature (>50°C) were recorded.

Ex vivo lesion assessment

Tissue was stained with 2% triphenyl tetrazolium chloride (TTC) in phosphate-buffered saline at 37°C. Following the initial staining, the myocardium was cross-sectioned at the level of each lesion and stained for a further 20mins. Lesion volume was calculated as previously described.[2] Briefly, the maximum depth (A), maximum diameter (B), depth at the maximum diameter (C), and lesion surface diameter (D), were measured for each lesion using Digital Vernier calipers (**Supplementary Figure 2C**). The lesion volume was calculated as: Lesion Volume = $(1/6)\pi*(A*B^2+C*D^2/2)$.[8] Tissues were fixed in 4% PFA and imaged under a dissecting microscope. Ablation sparing was measured using ImageJ (ImageJ; U.S. National Institutes of Health; http://rsbweb.nih.gov/ij/) software.

In vivo lab setup

Studies were performed using four healthy swine (German Landrace x Pietrain; 31–41kg (mean 36kg), 3-4 months old) at an accredited animal laboratory equipped with a Rhythmia HDx (Boston Scientific, Marlborough, MA) electroanatomic mapping system (Zentrum für Präklinische Forschung, Klinikum rechts der Isar, Technische Universität München, Munich, Germany). Electrophysiological studies were performed in vivo under general anaesthesia and mechanical ventilation during intrinsic sinus rhythm. Sedation was administered intramuscularly with ketamine (10-15mg/kg), azaperone (2mg/kg), and atropine (1mg). Induction of anaesthesia was with 1% and maintenance with 2% intravenous (IV) propofol. Acetylsalicylic acid (250mg IV) and heparin (150 IU/kg IV as a bolus and 200 IU/ml IV as a continuous drip infusion depending on activated clotting time) were administered for intraoperative anticoagulation after the sheath had been placed. Intraoperative analgesia was provided by fentanyl boluses (0.015mg/kg IV) every 20-30 min and metamizole (40-50mg/kg IV) was administered before the first incision. Transvenous catheters were inserted under fluoroscopic guidance. Limited electroanatomical mapping of the right atrium, right ventricle or both was performed in each animal during sinus rhythm using the ultra-high-density mapping system Rhythmia (Boston Scientific, Marlborough, MA) and its proprietary, 64-lead, multi-electrode basket mapping catheter Intellamap Orion (Boston Scientific, Marlborough, MA). Dosage of sedation was held constant within the whole period of mapping to ensure comparable conditions between the different maps. In one animal, access to the left heart was gained via transseptal puncture using a non-steerable sheath and a transseptal needle.

In vivo ablation protocol

Animal 1: 5 lesions in right ventricular tissue using an Intellanav Stablepoint (Boston Scientific, Marlborough, MA) open-irrigated catheter for Protocol A30 and Protocol B. All lesions were performed in power-controlled mode at 31W for 30s, aiming for a contact force of 10g.

Animals 2-4: 2-3 lines of ablation consisting of 7-10 lesions were performed in the right atrium and right ventricle for each of the following protocols: 1) Fixed irrigation at 30ml/min with centre-to-centre lesion spacing of 8mm. 2) The Protocol B with centre-to-centre lesion spacing of 8mm. 3) Fixed irrigation at 30ml/min with centre-to-centre lesion spacing of 5mm. For all animals, a Rhythmia mapping system was used to space lesions at least 15mm apart to ensure there was no overlap of lesions. Catheter tip temperature, starting and final bipolar electrogram amplitude, starting local impedance and local impedance drop were recorded.

Statistical Analysis

Parametric data are presented as mean ± SD. Lesion measurements were compared within and between groups by a Kruskal-Wallis test or one-way ANOVA (using Bonferroni's method for pairwise multiple comparisons) as appropriate. A value of $P<0.05$ was considered statistically significant.

Ethics

All studies were performed in accordance with the Guidance on the Operation of the Animals (Scientific Procedures) Act, 1986 (UK Home Office), and approved by the local ethics committee Newcastle University (Animal Welfare & Ethical Review Body). The *in vivo* study (Technical University Munich, Germany) was approved by the government of Bavaria (ROB-55.2-2532.Vet_02-1 7-174).
